# Beyond Pigmentation: Dermoscopic Vascular Patterns Reveal Novel Risk and Protective Factors in Melasma

**DOI:** 10.1111/jocd.71177

**Published:** 2026-10-25

**Authors:** Zhenghui Yang, Jing Wu, Qiuyan Yao, Hua Gu, Ying Tu

**Affiliations:** ^1^ Department of Dermatology and Venereology First Affiliated Hospital of Kunming Medical University Kunming China; ^2^ Baoshan Prefecture Center for Disease Control and Prevention Baoshan City China

**Keywords:** melasma, pigmentation, risk factors, telangiectasia, vascular dilation

## Abstract

**Objective:**

To investigate the correlation between the dermoscopic severity of telangiectasia and key pathophysiological features in melasma, and to identify the risk factors associated with vascular pattern severity.

**Methods:**

In this cross‐sectional study, 137 female melasma patients were classified into four vascular types based on dermoscopic evaluation: nonvascular change, erythema, strip‐ or branch‐shaped, and reticular. Comprehensive assessments included noninvasive skin measurements (Melanin Index [MI], Erythema Index [EI]), facial imaging, and detailed questionnaires. Multivariable ordinal logistic and multiple linear regression models were used for analysis.

**Results:**

The severity of the vascular type was significantly correlated with erythema parameters (*r* = 0.69) and the modified Melasma Area and Severity Index (mMASI). After multivariable adjustment, higher parity (Adjusted Odds Ratio [aOR] = 2.60) and higher Body Mass Index (BMI) (aOR = 1.38) emerged as the strongest independent risk factors for a more severe vascular type. In a separate model for mMASI, MI was the most potent positive predictor (*p* < 0.001), while intriguingly, erythema area ratio was a significant negative predictor (*p* = 0.026) after adjusting for MI.

**Conclusion:**

The severity of dermoscopic vascular patterns in melasma is strongly associated with increased erythema and clinical severity. Higher parity and BMI are key independent risk factors for severe telangiectasia. Our findings highlight the crucial role of the vascular component in melasma and suggest that clinical scoring systems may underestimate the contribution of erythema when significant pigmentation is present.

## 1 Introduction

Melasma is a common, chronic, and acquired hyperpigmentary disorder characterized by symmetric, light‐to‐dark brown macules and patches on sun‐exposed areas of the face. With a global prevalence ranging from 5% to 46% [1], melasma is a refractory and recurrent condition that significantly impacts patients' appearance and psychological well‐being [2]. While melasma can affect all skin types, it is most common in individuals with Fitzpatrick skin types III–IV [3], particularly in Asian populations. The pathogenesis of melasma is multifactorial, with established triggers including genetic predisposition, ultraviolet (UV) radiation, hormonal fluctuations, and heat exposure [1]. Epidemiological studies have demonstrated a positive correlation between melasma severity and factors such as age, duration of sun exposure, and heat from cooking or occupational sources [1]. Hormonal influences are particularly prominent, with a high incidence during pregnancy and a significant number of cases linked to the use of hormonal contraceptives [4], highlighting the crucial role of estrogen in its pathogenesis.

The Chinese Expert Consensus on Melasma (2021) has expanded the understanding of melasma beyond a simple disorder of melanogenesis. It is now conceptualized as a manifestation of photoaging, involving a complex interplay of pigmentary disturbances, epidermal barrier dysfunction, inflammation, and vascular proliferation. Melasma lesions exhibit a more pronounced increase in TEWL following barrier disruption compared to normal skin, coupled with a significantly delayed barrier recovery rate which indicates that melasma‐affected skin is characterized by compromised stratum corneum integrity and impaired barrier repair functions [5].

However, while the presence of vascular changes is acknowledged, a detailed classification of these vascular patterns and a comprehensive analysis of their correlation with pigmentation severity are still lacking. Furthermore, the specific risk factors that contribute to the development and severity of different vascular patterns in melasma remain largely unexplored.

Therefore, this study aims to fill these knowledge gaps. We propose a refined dermoscopic classification system for melasma based on four distinct vascular patterns: nonvascular change, erythema, strip‐ or branch‐shaped, and reticular. Using this classification, we will investigate the impact of varying degrees of telangiectasia on the severity of pigmentation and other pathophysiological features. Additionally, through a detailed questionnaire and objective measurements, we will analyze the risk and protective factors associated with these specific vascular patterns. This research seeks to provide a theoretical basis for the individualized prevention and treatment of melasma, as well as for targeted patient education.

## 2 Methods

### 2.1 Study Design and Participants

This cross‐sectional study was conducted at the Department of Dermatology and Veronology, the First Affiliated Hospital of Kunming Medical University, from September 2023 to April 2025. Female patients aged 18–60 years, diagnosed with melasma according to the 2003 diagnostic criteria by the Pigmentary Disorders Group of the Chinese Society of Dermatology, were included. All participants provided written informed consent before enrollment.

Exclusion criteria were as follows: (1) presence of severe systemic diseases involving the heart, kidney, liver, or endocrine system, as well as tumors or immunodeficiency disorders; (2) current pregnancy, planning for pregnancy, or breastfeeding; (3) concurrent facial pigmentary disorders that could interfere with the diagnosis, such as post‐inflammatory hyperpigmentation (e.g., from solar dermatitis, facial eczema, or contact dermatitis), nevus of Ota, Riehl's melanosis, pigmentary lichen planus, Addison's disease, Civatte's poikiloderma, or drug‐induced facial pigmentation.

### 2.2 Dermoscopic Classification

All participants underwent dermoscopic examination of their facial lesions. Based on the observed erythema and telangiectasia patterns, patients were classified into four distinct vascular types: nonvascular change, erythema, strip‐ or branch‐shaped, and reticular.

### 2.3 Questionnaire Survey

A structured questionnaire was developed based on a literature review of Chinese and international databases using keywords such as “melasma,” “trigger factors,” and “pathogenesis.” The questionnaire was administered by a professional dermatologist and collected data on three main dimensions:

1. General Information: Age, Body Mass Index (BMI), marital status, education level, occupation type, and Fitzpatrick skin type.
2. Clinical History: Age of onset, disease duration, treatment history, menstrual and obstetric history, and use of oral contraceptives.
3. Daily Lifestyle Habits: Circadian rhythm, assessed using the Morningness‐Eveningness Questionnaire (MEQ); duration of indoor/outdoor activities, electronics use, and heat exposure, both before and after the onset of melasma.

### 2.4 Objective Skin Assessments

A comprehensive panel of noninvasive instrumental measurements was performed on the lesional skin of each participant.

1. Skin Physiological Function: A multi‐probe skin testing device was used to measure Trans‐Epidermal Water Loss (TEWL), Melanin Index (MI), Erythema Index (EI), and Skin Cuticle Hydration (SCH).
2. Skin Structure and Reactivity: Skin thickness was measured by Reflectance Confocal Microscopy (RCM). Skin sensitivity and vascular reactivity were evaluated using the Lactic Acid Stinging Test (LAST) and a capsaicin test (CAT). The Transient Irritation Vascular Index (TIVI) was measured with a skin sensitivity meter to quantify the vascular response to these stimuli.

### 2.5 Clinical Scoring and Classification

The severity of melasma was scored using the modified Melasma Area and Severity Index (mMASI), calculated based on photographs taken under standard and Wood's lamp illumination. Participants' skin phototype was classified according to the Fitzpatrick skin typing system.

### 2.6 Statistical Analysis

All statistical analyses were performed using SPSS version 27.0 (IBM Corp., Armonk, NY, USA). A two‐tailed *p*‐value < 0.05 was considered statistically significant. Continuous variables were tested for normality using the Shapiro–Wilk test. Normally distributed data were presented as mean ± standard deviation (SD) and compared among groups using one‐way analysis of variance (ANOVA). Non‐normally distributed data were presented as median and interquartile range (IQR) and compared using the Kruskal–Wallis H test. Categorical variables were expressed as frequency and percentage (*n*, %) and compared using the Chi‐square (*χ* ^2^) test or Fisher's exact test, as appropriate. Spearman correlation analysis was used to assess monotonic relationships between variables. To identify the independent risk factors for the severity of the vascular type (the primary outcome), a multivariable ordinal logistic regression model was constructed. The proportional odds assumption was checked using the test of parallel lines. Furthermore, to explore the independent predictors of melasma severity, a multiple linear regression model was developed with the mMASI score as the dependent variable. The assumptions of linear regression, including the normality of residuals, were checked. To build our regression models, we employed a principled, multistep approach. First, we conducted a broad univariate screening to identify all potential candidate variables, using a liberal inclusion threshold of *p* < 0.10. Next, to address potential multicollinearity, we examined a Spearman correlation matrix of these candidates and excluded any variables that were conceptually redundant (e.g., retaining BMI over weight). The final set of selected variables was then entered into the multivariable model. For the final linear regression model, we further checked for multicollinearity using the variance inflation factor (VIF).

To identify the independent factors associated with the severity of the vascular type (the primary outcome), a multivariable ordinal logistic regression model was constructed. The proportional odds assumption was checked using the test of parallel lines. Furthermore, to explore the independent predictors of melasma severity, a multiple linear regression model was developed with the mMASI score as the dependent variable; the assumptions of linear regression, including the normality of residuals, were verified.

## 3 Results

### 3.1 Baseline Characteristics and Vascular Type Distribution

A total of 151 patients with melasma were enrolled in this study. After excluding those with incomplete data, 137 patients with valid data were included in the final analysis. The mean age of the participants was 38.13 ± 8.91 years. Based on dermoscopic findings, the patients were classified into four vascular types: the nonvascular change type (*n* = 17, Figures 1a and 2), the erythema type (*n* = 31, Figures 1b and 2), the strip‐ or branch‐shaped vascular type (*n* = 57, Figures 1c and 2), and the reticular vascular type (*n* = 32, Figures 1d and 2).

**FIGURE 1:**
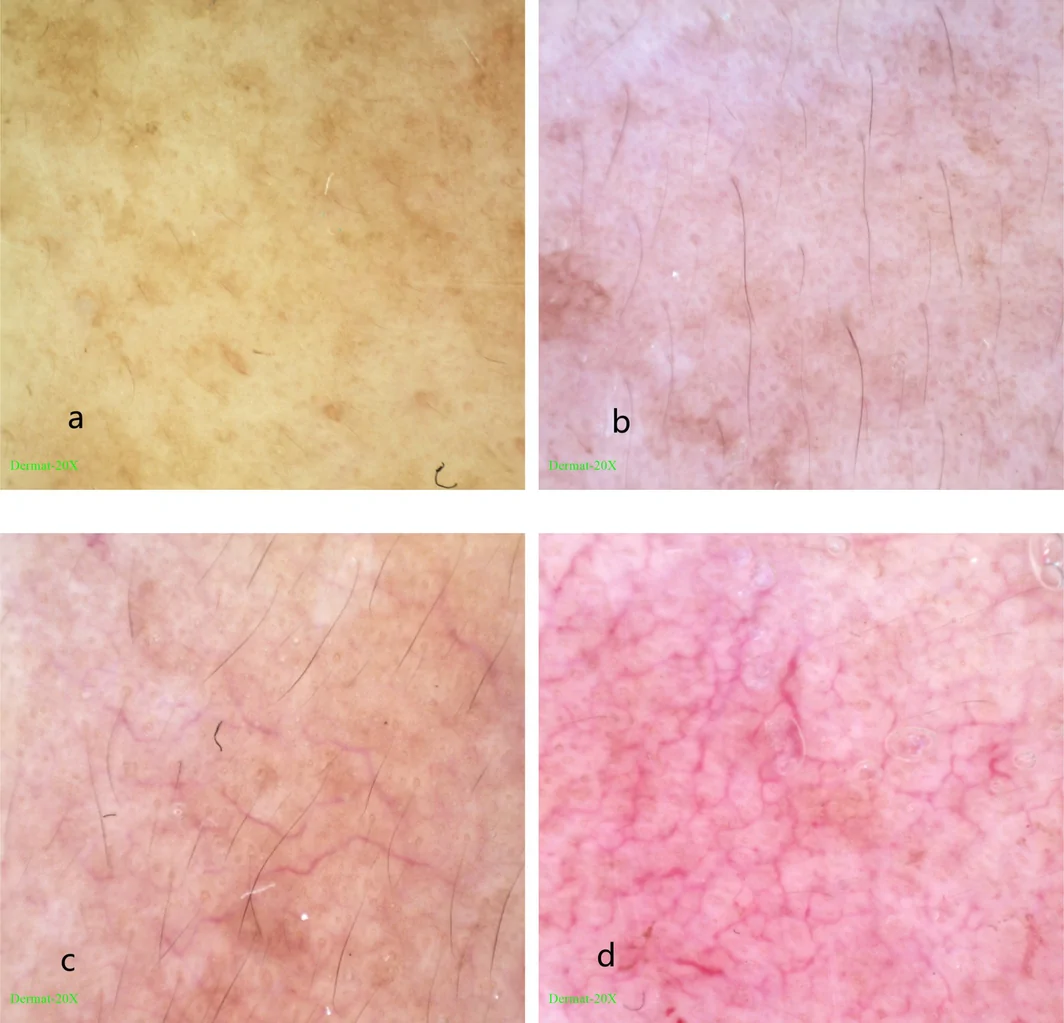
Dermoscopic features of the four vascular morphologies in melasma: (a) nonvascular change, (b) erythema, (c) strip‐ or branch‐shaped, and (d) reticular.

**FIGURE 2:**
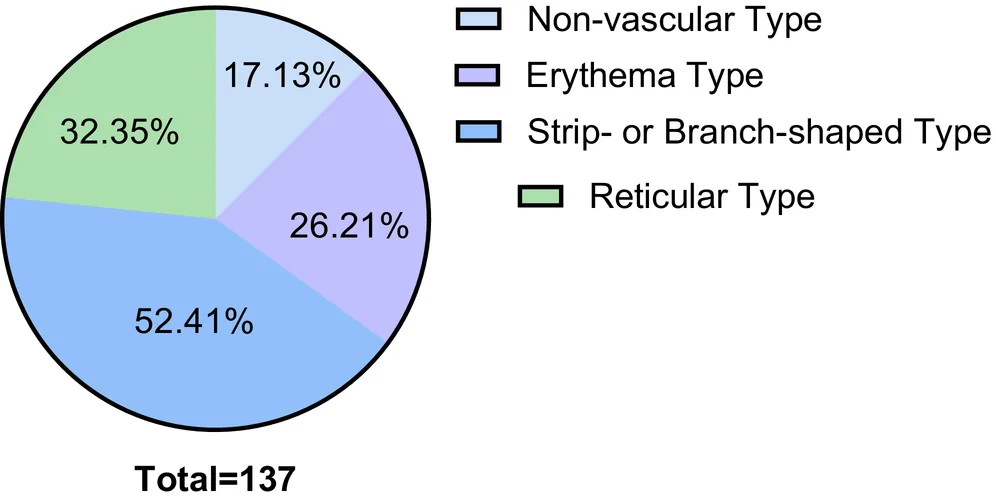
Distribution of the four dermoscopic vascular types among the study participants (*n* = 137).

### 3.2 Comparison of Skin Physiological Vascular Parameters and Structural Parameters Among Different Melasma Vascular Types

To investigate the relationship between the severity of telangiectasia and the pathophysiological characteristics of melasma, we conducted group comparisons, correlation analyses, and univariate ordinal logistic regressions.

Group comparisons revealed that the Reticular Type exhibited a significantly higher Melanin Index (MI) compared to the other three groups (*p* < 0.0001, Figure 3A). A clear trend was also observed for the mMASI score, which progressively increased with the severity of telangiectasia (Figure 3B); specifically, the Reticular Type showed a significantly higher mMASI score than the other three types (*p* = 0.0028, Figure 3B). The correlation analysis provided further insights, showing that the vascular type was positively correlated with both mMASI (*r* = 0.29) and MI (*r* = 0.13) (Figure 4). Notably, MI was also moderately correlated with the Erythema Index (EI) (*r* = 0.40) (Figure 4), suggesting a link between vascular activity and pigmentation. When these factors were assessed as predictors for vascular severity in univariate ordinal logistic regression, mMASI emerged as a significant risk factor (OR = 1.12, *p* = 0.003, Table 1), indicating that a higher clinical severity score is associated with a higher risk of more severe vascular patterns. In contrast, the MI value did not show a statistically significant association (OR = 1.00, *p* = 0.209, Table 1).

**FIGURE 3:**
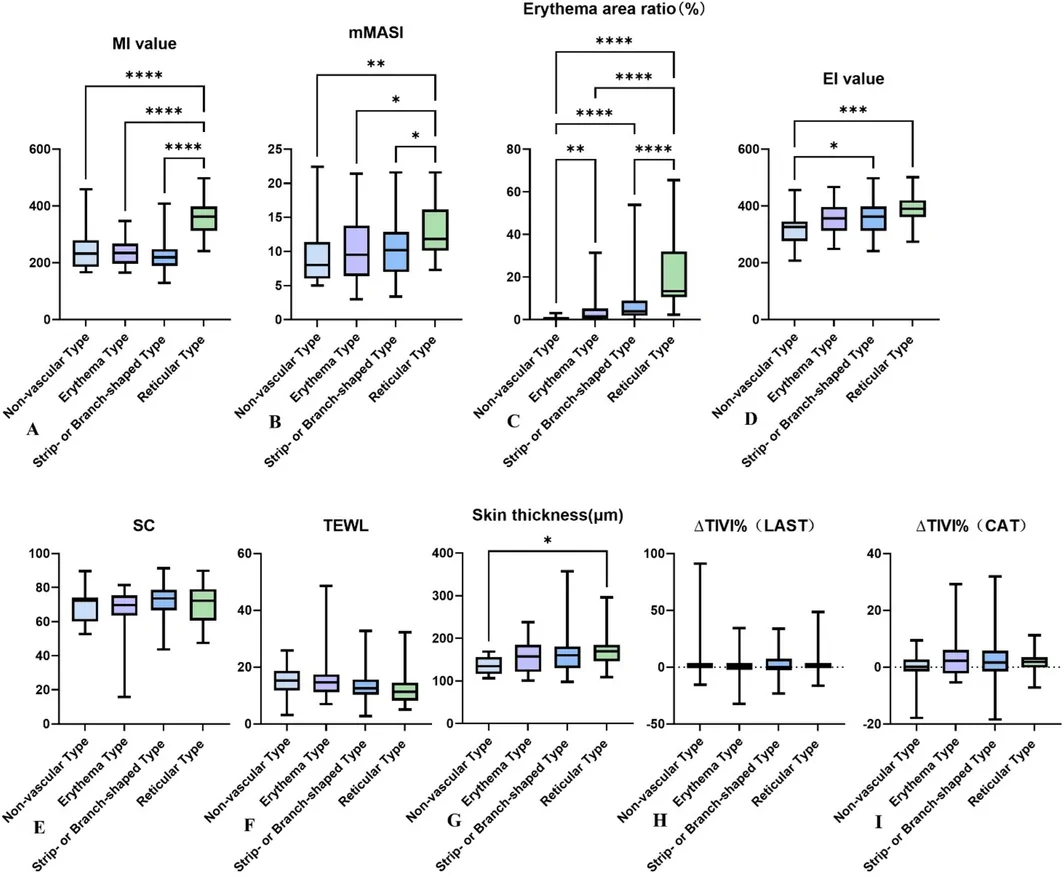
Comparison of objective skin parameters among the four vascular types. The box plots display the (A) Melanin Index (MI), (B) mMASI score, (C) Erythema Area Ratio, (D) Erythema Index (EI), (E) skin cuticle hydration (SC), (F) trans‐epidermal water loss (TEWL), (G) skin thickness (μm), (H) ∆TIVI% (LAST), and (I) ∆TIVI% (CAT). Data are presented as median (Q1, Q3). Statistical significance was determined by one‐way ANOVA or Kruskal–Wallis test, followed by post hoc tests. Significant differences are indicated by asterisks (*p < 0.05, **p < 0.01, ***p < 0.001, ****p < 0.0001).

**FIGURE 4:**
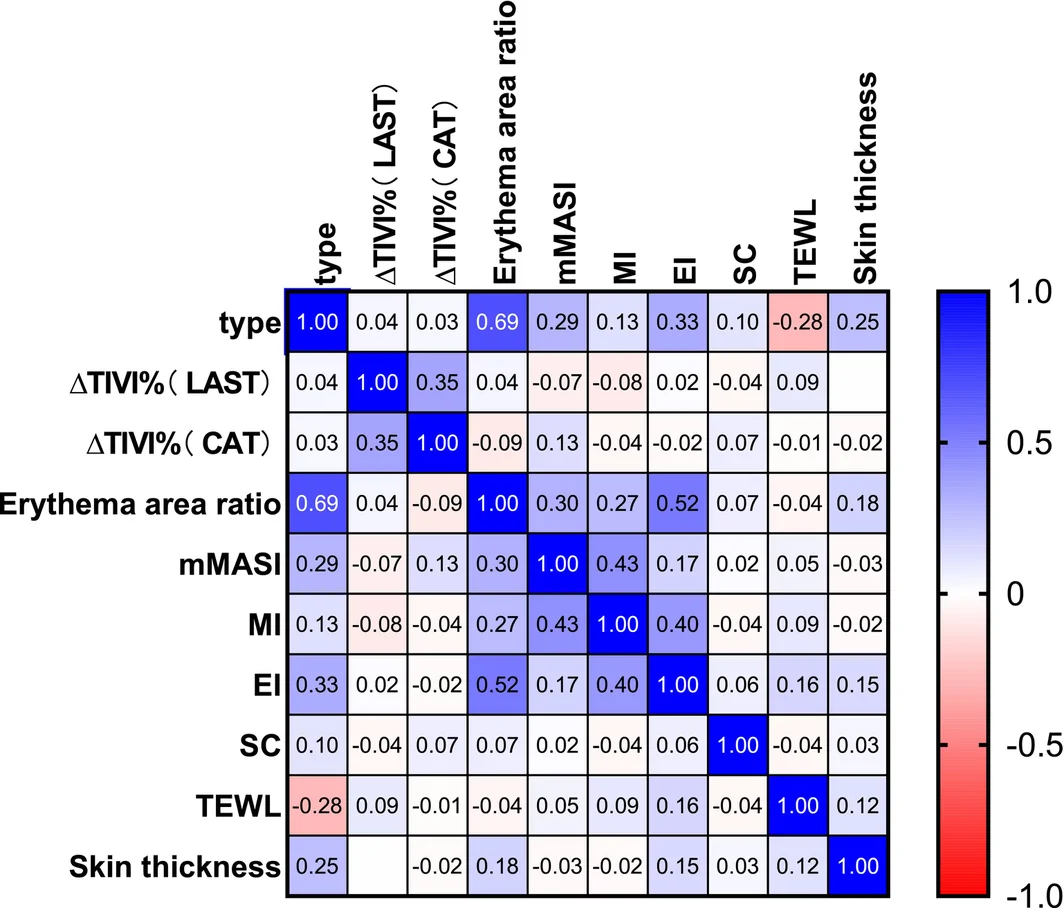
Heatmap of the Spearman correlation matrix for vascular type and objective skin parameters. The matrix visualizes the Spearman's rank correlation coefficient (*r*) between all pairs of variables. The color intensity and the number in each cell represent the strength and direction of the correlation, as indicated by the color scale on the right. Blue cells indicate a positive correlation, while red cells indicate a negative correlation. White cells represent correlations that were not statistically significant (*p* ≥ 0.05).

**TABLE 1:** Association between the grade of telangiectasia and the severity of pigmentation [median (Q1, Q3)].

Variable and category	Nonvascular type	Erythema type	Strip‐ or branch‐shaped type	Reticular type	Group comparison *χ* ^2^ (*p*)	Univariate ordinal logistic regression OR (95% CI, *p*)
MI value	232.67 (187.32, 269.32)	234 (197.42, 263.67)	219.34 (191.15, 246.77)	253.67 (225.88, 286.75)	89.7 (< 0.0001)	1.00 (1.00–1.01, 0.209)
mMASI	8.0 (6.2, 10.8)	9.5 (7.15, 13.6)	10.2 (7.1, 12.8)	11.85 (10.18, 16.05)	14.11 (0.0028)	1.12 (1.04–1.20, 0.003)

In terms of erythema, both the Erythema Area Ratio and the EI value showed a stepwise increase corresponding to the grade of vascular dilation. The differences in both Erythema Area Ratio (*p* < 0.0001, Figure 3C) and EI value (*p* = 0.0007, Figure 3D) among the four groups were statistically significant. The link between vascular severity and erythema was powerfully underscored by the correlation analysis, which showed strong positive correlations between the vascular type and both the Erythema Area Ratio (*r* = 0.69) and the EI value (*r* = 0.33) (Figure 4). Further validating these findings, both erythema parameters were identified as significant risk factors for more severe vascular types in the univariate ordinal logistic regression. The Erythema Area Ratio (OR = 1.12, *p* < 0.001) and the EI value (OR = 1.01, *p* < 0.001) were both independently associated with an increased risk of a higher vascular grade (Table 2).

**TABLE 2:** Relationship between erythema indices and the severity of telangiectasia [Median (Q1, Q3)].

Variable and category	Nonvascular type	Erythema type	Strip‐ or branch‐shaped type	Reticular type	Group comparison *χ* ^2^ (*p*)	Univariate ordinal logistic regression OR (95% CI, *p*)
Erythema area ratio	0.13% (0.05%, 0.27%)	1.52% (0.47%, 5.12%)	3.92% (1.88%, 8.54%)	13.43% (11.06%, 30.49%)	67.86 (< 0.0001)	1.12 (1.07–1.16, < 0.001)
EI value	325.5 (277.08, 340.47)	356 (313.92, 394.79)	362.84 (317.85, 397.17)	390.5 (362.84, 418.13)	16.99 (0.0007)	1.01 (1.00–1.02, < 0.001)
∆TIVI% (LAST)	0.11% (−0.97%, 2.81%)	0.36% (−1.75%, 3.23%)	−0.12% (−2.31%, 7.07%)	1.49% (−0.42%, 3.24%)	0.2054 (0.9767)	0.56 (0.05–6.37, 0.636)
∆TIVI% (CAT)	0.08% (−0.85%, 2.55%)	2.28% (−2.01%, 5.56%)	1.62% (−1.43%, 5.48%)	1.84% (0.27%, 3.46%)	1.912 (0.5908)	0.99 (0.02–62.12, 0.994)

The relationship between vascular severity, skin barrier, and structure presented an intriguing paradox. Group comparisons showed that Trans‐Epidermal Water Loss (TEWL) was highest in the Erythema Type and lowest in the Reticular Type, with the overall difference being statistically significant (*p* < 0.05, Figure 3F). In contrast, skin thickness significantly increased with severity, with the Reticular Type being thicker than the Nonvascular Type (*p* < 0.05, Figure 3G). Correlation analysis supported these findings, revealing a significant positive correlation between vascular type and skin thickness (*r* = 0.25) and a significant negative correlation with TEWL (*r* = −0.28) (Figure 4).

The logistic regression analysis provided a potential resolution to this paradox. While skin thickness and Skin Cuticle Hydration (SC) were not significant predictors, TEWL was identified as a significant protective factor (OR = 0.94, *p* = 0.02, Table 3).

**TABLE 3:** Comparison of skin barrier function and structural parameters among different Melasma vascular types [median (Q1, Q3)].

Variable and category	Nonvascular type	Erythema type	Strip‐ or branch‐shaped type	Reticular type	Group comparison *χ* ^2^ (*p*)	Univariate ordinal logistic regression OR (95% CI, *p*)
SC	72.23 (61.19, 74.15)	69.97 (63.7, 75.17)	73.6 (67.64, 78.67)	72.27 (61.20, 78.80)	4.521 (0.2104)	1.02 (0.99–1.04, 0.259)
TWEL	15.3 (13.05, 18.4)	14.6 (11.3, 17.3)	12.6 (10.4, 15.4)	11.35 (8.28, 13.9)	9.918 (0.0193)	0.94 (0.90–0.99, 0.02)
Skin thickness (μm)	134.44 (119.90, 153.82)	157.58 (122.10, 182.79)	160.26 (131.14, 184.61)	169.48 (150.39, 3.46%)	9.764 (0.0207)	1.00 (1.00–1.00, 0.852)

Finally, both group comparisons and regression analyses confirmed that vascular reactivity parameters ∆TIVI%^1^ (LAST and CAT) were not significantly associated with the severity of telangiectasia (*p* > 0.05, Table 2, Figure 4).

### 3.3 Univariate Analysis of Factors Associated With Telangiectasia Severity

To identify factors associated with the severity of telangiectasia, univariate analyses including group comparisons and ordinal logistic regressions were performed. The detailed results are presented in Tables 4, 5, 6, 7, 8.

**TABLE 4:** Comparison of demographic and lifestyle factors among different melasma vascular types [median (Q1, Q3)].

Variable and category	Nonvascular change type	Erythema type	Strip‐ or branch‐shaped vascular type	Reticular vascular type	Group comparison *χ* ^2^ (*p*)	Univariate ordinal logistic regression OR (95% CI, *p*)
Age (years)	35 (31, 41)	34 (29, 39)	39 (30, 45)	41 (36, 47)	12.72 (0.005)	1.06 (1.02–1.10, 0.003)
BMI (kg/m^2^)	20.0 (19.1, 21.3)	19.9 (19, 21.1)	20.8 (20.0, 22.8)	22.7 (21.5, 23.8)	22.15 (< 0.0001)	1.38 (1.19–1.59, < 0.001)
Number of pregnancies	2 (1, 1)	2 (2, 3)	2 (2, 3)	2 (2, 4)	0.794 (0.5)	1.22 (0.92, 1.61, 0.159)
Number of abortions	0 (0, 1)	1 (1, 2)	1 (0, 2)	1 (0, 1)	0.275 (0.843)	0.93 (0.67–1.30, 0.67)
Number of deliveries	1 (1, 2)	1 (1, 2)	1 (1, 2)	2 (1, 2)	12.98 (0.0047)	2.60 (1.40–4.82, 0.002)
Disease duration (years)	7 (5, 10)	4 (3, 7)	6 (3, 10)	8 (5, 11.5)	8.386 (0.0387)	1.05 (1.01–1.11, 0.029)
MEQ	48 (47, 51)	49 (43.5, 53)	49 (46, 55)	55 (49, 58)	10.48 (0.0149)	1.09 (1.01–1.17, 0.022)
Electronics use (before onset, h/day)	5.5 (3.5, 8)	6.5 (4.5, 8)	6.5 (3, 8)	3.5 (1.9, 8)	6.351 (0.0957)	0.89 (0.81–0.98, 0.02)
Electronics use (after onset, h/day)	7.5 (5.5, 10)	7.5 (5.5, 9.25)	7.5 (4.5, 10)	4.5 (3, 8, 1)	8.561 (0.0357)	0.86 (0.78–0.95, 0.004)
Kitchen exposure (before onset, h/day)	0.5 (0, 1)	0.5 (0, 1)	0.5 (0, 1)	1 (0, 2.5)	8.858 (0.0312)	1.75 (1.25–2.43, < 0.001)
Kitchen exposure (after onset, h/day)	0.5 (0, 1)	0.5 (0, 1)	0.5 (0, 1)	1 (0, 2.1)	8.932 (0.0302)	1.75 (1.25–2.43, 0.001)

Significant differences among the four vascular types were found in the distribution of age (*p* = 0.005, Figure 5A, Table 4), BMI (*p* < 0.001, Figure 5B, Table 4), parity (*p* = 0.0047, Figure 5C, Table 4), disease duration (*p* = 0.0387, Figure 5D, Table 4), MEQ score (*p* = 0.0149, Figure 5E, Table 4), marital status (*p* = 0.006, Table 5), and education level (*p* = 0.005, Table 5). Specifically, a trend of increasing median age, BMI, parity, disease duration, and MEQ score was observed with increasing severity of telangiectasia (Figure 5). The proportion of patients with a lower education level (Below Bachelor) was highest in the Reticular type group (Table 5).

**FIGURE 5:**
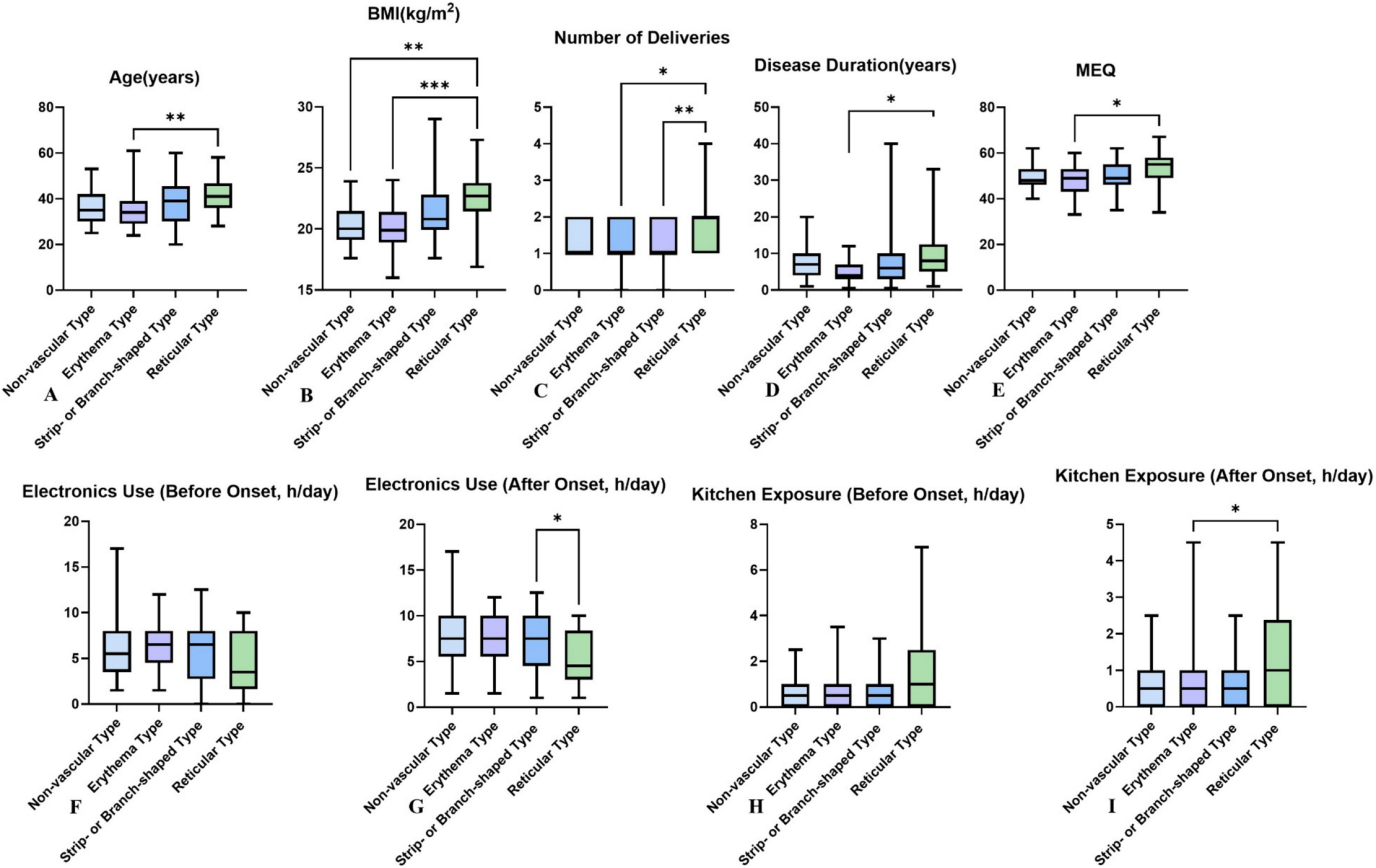
Comparison of demographic and lifestyle factors among the four vascular types. The box plots display the (A) Age (years), (B) BMI (kg/m^2^), (C) number of deliveries, (D) disease duration (years), (E) MEQ score, (F) pre‐onset electronics use (h/day), (G) post‐onset electronics use (h/day), (H) pre‐onset kitchen exposure (h/day), and (I) post‐onset kitchen exposure (h/day). Data are presented as median [Q1, Q3]. Statistical significance was determined by one‐way ANOVA or Kruskal–Wallis test, followed by post hoc tests. Significant differences are indicated by asterisks (*p < 0.05, **p < 0.01, ***p < 0.001).

**TABLE 5:** Comparison of personal history among the four groups.

Variable and category	Nonvascular type	Erythema type	Strip‐ or branch‐shaped type	Reticular type	Group comparison *χ* ^2^ (*p*)	Univariate ordinal logistic regression OR (95% CI, *p*)
*Marital status*					12.418 (0.006)	0.36 (0.18–0.73, 0.005)
Unmarried	5 (29.4%)	14 (45.2%)	15 (26.3%)	2 (6.2%)		
Married	12 (70.6%)	17 (54.8%)	42 (73.7%)	30 (93.8%)		
*History of pregnancy*					7.208 (0.066)	0.44 (0.22–0.87, 0.018)
No	6 (35.29%)	14 (45.16%)	15 (26.32%)	5 (15.63%)		
Yes	11 (64.71%)	17 (54.84%)	42 (73.68%)	27 (84.38%)		
*Menstrual regularity*					4.115 (0.249)	1.57 (0.82–3.01, 0.173)
Irregular	6 (35.29%)	6 (19.35%)	22 (38.60%)	13 (40.63%)		
Regular	11 (64.71%)	25 (80.65%)	35 (61.40%)	19 (59.38%)		
*Menstrual volume*					3.969 (0.265)	0.77 (0.42–1.42, 0.4)
Normal	5 (29.41%)	17 (54.84%)	32 (56.14%)	16 (50.00%)		
Abnormal	12 (70.59%)	14 (45.16%)	25 (43.86%)	16 (50.00%)		
*MEQ type*					7.457 (0.281)	
Evening type	1 (5.88%)	6 (19.35%)	4 (7.02%)	1 (3.13%)		0.26 (0.06–1.10, 0.067)
Intermediate type	15 (88.24%)	23 (74.19%)	47 (82.46%)	26 (81.25%)		0.53 (0.19–1.49, 0.229)
Morning type	1 (5.88%)	2 (6.45%)	6 (10.53%)	5 (15.63%)		
*Fitzpatrick*					3.526 (0.317)	1.75 (0.84–3.64, 0.135)
TypeIII	13 (76.47%)	21 (67.74%)	44 (77.19%)	28 (87.50%)		
Type IV	4 (23.53%)	10 (32.26%)	13 (22.81%)	4 (12.50%)		
*Change of residence*					6.848 (0.077)	1.95 (1.02–3.73, 0.043)
No	7 (41.18%)	21 (67.74%)	36 (63.16%)	25 (78.12%)		
Yes	10 (58.82%)	10 (32.26%)	21 (36.84%)	7 (21.88%)		
*Family history*					5.652 (0.13)	1.37 (0.74–2.53, 0.318)
No	9 (52.94%)	12 (38.71%)	37 (64.91%)	17 (53.12%)		
Yes	8 (47.06%)	19 (61.29%)	20 (35.09%)	15 (46.88%)		
*Education level*					12.796 (0.005)	3.03 (1.47–6.27, 0.003)
Below bachelor	2 (11.8%)	7 (22.6%)	11 (19.3%)	16 (50.0%)		
Bachelor or above	15 (88.2%)	24 (77.4%)	46 (80.7%)	16 (50.0%)		

Consistent with these findings, univariate ordinal logistic regression identified a lower education level (OR = 3.03, Table 5), higher parity (OR = 2.60, Table 4), higher BMI (OR = 1.38, Table 4), older age (OR = 1.06, Table 4), longer disease duration (OR = 1.05, Table 4), and a higher MEQ score (OR = 1.07, Table 4) as significant risk factors. Conversely, being unmarried (OR = 0.36, Table 5) and having no history of pregnancy (OR = 0.44, Table 5) were significant protective factors. The number of pregnancies and abortions, as well as menstrual characteristics and Fitzpatrick skin type, showed no significant differences among the groups (*p* > 0.05, Tables 4 and 5).

Group comparisons revealed significant differences in the distribution of before‐ and after‐onset kitchen exposure (*p* = 0.031 and *p* = 0.030, respectively, Figure 5H,I, Table 4) and before‐ and after‐onset heat exposure (*p* = 0.005 and *p* = 0.031, respectively, Table 6). Group comparisons revealed significant differences in the distribution of heat exposure and the median duration of kitchen exposure (*p* < 0.05 for both; Tables 4 and 6). Specifically, the proportion of patients with prolonged heat exposure (> 1 h/day) was progressively higher in the more severe vascular types. A similar trend was observed for kitchen exposure, where the median duration of daily exposure increased with the severity of the vascular pattern. Logistic regression confirmed that both prolonged kitchen exposure (before onset, OR = 1.75) and heat exposure (before onset, OR = 3.13) were strong risk factors (Tables 4 and 6).

**TABLE 6:** Relationship between telangiectasia and daily lifestyle habits.

Variable and category	Nonvascular type	Erythema type	Strip‐ or branch‐shaped type	Reticular type	Group comparison *χ* ^2^ (*p*)	Univariate ordinal logistic regression OR (95% CI, *p*)
*Heat exposure (before onset)*					12.871 (0.005)	0.32 (0.15–0.68, 0.003)
≤ 1 h/day	15 (88.24%)	24 (80.00%)	47 (83.93%)	17 (53.13%)		
> 1 h/day	2 (11.76%)	6 (20.00%)	9 (16.07%)	15 (46.88%)		
*Heat exposure* (*after onset*)					8.857 (0.031)	0.39 (0.19–0.79, 0.01)
≤ 1 h/day	14 (82.35%)	24 (80.00%)	44 (78.57%)	17 (53.13%)		
> 1 h/day	3 (17.65%)	6 (20.00%)	12 (21.43%)	15 (46.88%)		
*Outdoor exposure* (*before onset*)					7.16 (0.067)	1.90 (1.02–3.53, 0.043)
≤ 2 h/day	6 (35.29%)	14 (45.16%)	38 (66.67%)	18 (56.25%)		
> 2 h/day	11 (64.71%)	17 (54.84%)	19 (33.33%)	14 (43.75%)		
Outdoor exposure (after onset)					7.359 (0.061)	1.77 (0.94–3.35, 0.077)
≤ 2 h/day	8 (47.06%)	17 (54.84%)	45 (78.95%)	20 (62.50%)		
> 2 h/day	9 (52.94%)	14 (45.16%)	12 (21.05%)	12 (37.50%)		
*Use of curtains for sun protection (before onset*)					2.912 (0.405)	0.54 (0.23–1.28, 0.16)
No	16 (94.12%)	28 (90.32%)	46 (80.70%)	26 (81.25%)		
Yes	1 (5.88%)	3 (9.68%)	11 (19.30%)	6 (18.75%)		
*Use of curtains for sun protection (after onset*)					1.909 (0.591)	0.73 (0.35–1.54, 0.412)
No	13 (76.47%)	27 (87.10%)	43 (75.44%)	24 (75.00%)		
Yes	4 (23.53%)	4 (12.90%)	14 (24.56%)	8 (25.00%)		
*General sunscreen use (before onset)*					1.491 (0.684)	1.05 (0.57–1.94, 0.865)
No	10 (58.82%)	16 (51.61%)	27 (47.37%)	19 (59.38%)		
Yes	7 (41.18%)	15 (48.39%)	30 (52.63%)	13 (40.62%)		
*General sunscreen use (after onset)*					3.251 (0.354)	2.62 (0.86–7.99, 0.09)
No	1 (5.88%)	1 (3.23%)	5 (8.77%)	5 (15.63%)		
Yes	16 (94.12%)	30 (96.77%)	52 (91.23%)	27 (84.38%)		
*Chemical sunscreen use (before onset)*					1.171 (0.76)	1.11 (0.60–2.05, 0.749)
No	11 (64.71%)	16 (51.61%)	32 (56.14%)	20 (62.50%)		
Yes	6 (35.29%)	15 (48.39%)	25 (43.86%)	12 (37.50%)		
*Chemical sunscreen use (after onset)*					3.051 (0.384)	1.71 (0.73–3.97, 0.213)
No	3 (17.65%)	2 (6.45%)	10 (17.54%)	7 (21.88%)		
Yes	14 (82.35%)	29 (93.55%)	47 (82.46%)	25 (78.13%)		
*Physical sunscreen use (before onset)*					1.694 (0.638)	1.29 (0.69–2.43, 0.427)
No	11 (64.71%)	18 (58.06%)	34 (59.65%)	23 (71.88%)		
Yes	6 (35.29%)	13 (41.94%)	23 (40.35%)	9 (28.12%)		
*Physical sunscreen use (after onset)*					2.18 (0.536)	1.46 (0.69–3.28, 0.361)
No	2 (11.76%)	6 (19.35%)	8 (14.04%)	8 (25.00%)		
Yes	15 (88.24%)	25 (80.65%)	49 (85.96%)	24 (75.00%)		
*Combined (physical + chemical) sunscreen use*					0.168 (0.983)	1.12 (0.60–2.09, 0.729)
No	6 (35.29%)	12 (38.71%)	23 (40.35%)	13 (40.62%)		
Yes	11 (64.71%)	19 (61.29%)	34 (59.65%)	19 (59.38%)		
*Frequency of sunscreen application*					11.824 (0.066)	
≥ 1 time/day	13 (76.47%)	19 (61.29%)	31 (54.39%)	21 (65.62%)		2.07 (0.74–5.78, 0.166)
Occasionally per week	1 (5.88%)	11 (35.48%)	17 (29.82%)	4 (12.50%)		1.08 (0.52–2.24, 0.845)
Never	3 (17.65%)	1 (3.23%)	9 (15.79%)	7 (21.88%)		

For electronics use, a significant difference among groups was found for the after onset duration (*p* = 0.0357, Figure 5G, Table 4), with the Reticular type showing lower usage. Intriguingly, logistic regression identified longer electronics use as a protective factor, both before (OR = 0.89) and after onset (OR = 0.86) (Table 4).

For residence and outdoor exposure, the overall group distributions did not reach statistical significance (*p* > 0.05, Table 5). However, logistic regression revealed that not changing residence (OR = 1.95, *p* = 0.043, Table 5) was a significant risk factor, and before‐onset outdoor exposure > 2 h/day (OR = 0.53, *p* = 0.043, Table 6) was a significant protective factor.

The clinical course, including the timing of onset and period of exacerbation, showed significant associations with the severity of vascular types, as detailed in Table 7. A statistically significant difference was found in the distribution of timing of onset among the four groups (*p* = 0.022, Table 7). Specifically, the proportion of patients with a before pregnancy onset decreased with increasing vascular severity, whereas the proportion with a during pregnancy onset was highest in the more severe types. This was further supported by the ordinal logistic regression, which showed a nonsignificant trend for before pregnancy onset being a protective factor (OR = 0.51, *p* = 0.061, Table 7) and during pregnancy onset being a risk factor (OR = 2.27, *p* = 0.054, Table 7). For the period of exacerbation, while the overall group distribution was not statistically significant (*p* = 0.239, Table 7), a trend was observed where a higher proportion of patients in the postpartum category was found in the more severe vascular groups. Logistic regression also suggested a protective trend for exacerbation before pregnancy (OR = 0.48, *p* = 0.067, Table 7). In contrast, no statistically significant differences were found among the four vascular types regarding the history of contraceptive medication use, regular follow‐up visits, or the use of various treatments, including topical medications, whitening agents, chemical peels, laser or light therapy, and systemic medications (*p* > 0.05 for all, Table 7).

**TABLE 7:** Association of clinical characteristics with different types of melasma.

Variable and category	Nonvascular type	Erythema type	Strip‐ or branch‐shaped type	Reticular type	Group comparison *χ* ^2^ (*p*)	Univariate ordinal logistic regression OR (95% CI, *p*)
*Period of exacerbation*					11.558 (0.239)	
No change	4 (25.00%)	9 (30.00%)	16 (28.57%)	5 (15.63%)		0.62 (0.28–1.36, 0.232)
Before pregnancy	7 (43.75%)	9 (30.00%)	12 (21.43%)	6 (18.75%)		0.48 (0.22–1.05, 0.067)
During pregnancy	2 (12.50%)	2 (6.67%)	3 (5.36%)	7 (21.88%)		1.86 (0.62–5.57, 0.269)
Postpartum	5 (18.75%)	10 (33.33%)	25 (44.64%)	14 (43.75%)		
*Timing of onset*					14.82 (0.022)	
Before pregnancy	9 (52.94%)	17 (54.84%)	20 (35.09%)	7 (21.88%)		0.51 (0.25–1.03, 0.061)
During pregnancy	3 (17.65%)	1 (3.23%)	15 (26.32%)	12 (37.50%)		2.27 (0.99–5.23, 0.054)
Postpartum	5 (29.41%)	13 (41.94%)	22 (38.60%)	13 (40.63%)		
*Use of contraceptive medication*					3.028 (0.387)	0.90 (0.48–1.69, 0.745)
No	10 (58.82%)	23 (74.19%)	32 (56.14%)	21 (65.62%)		
Yes	7 (41.18%)	8 (25.81%)	25 (43.86%)	11 (34.38%)		
*Follow‐up visit*					0.583 (0.9)	1.01 (0.22–3.12, 0.982)
No	15 (88.24%)	29 (93.55%)	53 (92.98%)	29 (90.63%)		
Yes	2 (11.76%)	2 (6.45%)	4 (7.02%)	3 (9.38%)		
*Use of topical medications*					2.368 (0.5)	1.71 (0.84–3.51, 0.14)
No	11 (64.71%)	22 (70.97%)	45 (78.95%)	26 (81.25%)		
Yes	6 (35.29%)	9 (29.03%)	12 (21.05%)	6 (18.75%)		
*Use of whitening agents*					1.084 (0.781)	0.79 (0.43–1.48, 0.465)
No	12 (70.59%)	18 (58.06%)	33 (57.89%)	18 (56.25%)		
Yes	5 (29.41%)	13 (41.94%)	24 (42.11%)	14 (43.75%)		
*Use of chemical peels*					7.797 (> 0.05)	1.30 (0.64–2.64, 0.467)
No	14 (82.35%)	23 (74.19%)	37 (64.91%)	29 (90.63%)		
Yes	3 (17.65%)	8 (25.81%)	20 (35.09%)	3 (9.38%)		
*History of laser or light therapy*					4.202 (0.24)	1.71 (0.60–2.28, 0.643)
No	12 (70.59%)	23 (74.19%)	35 (61.40%)	26 (81.25%)		
Yes	5 (29.41%)	8 (25.81%)	22 (38.60%)	6 (18.75%)		
*Use of systemic medications*					0.234 (0.972)	1.04 (0.54–2.02, 0.909)
No	11 (64.71%)	22 (70.97%)	40 (70.18%)	22 (68.75%)		
Yes	6 (35.29%)	9 (29.03%)	17 (29.82%)	10 (31.25%)		

Given that parity was identified as one of the strongest risk factors in the univariate analysis, we constructed a series of multivariate models to reevaluate the independent effect of other key demographic and lifestyle factors after adjusting for its potent confounding influence (Table 8). After adjustment for parity, prolonged kitchen exposure remained a significant independent risk factor for more severe telangiectasia, both before (aOR = 1.52, 95% CI: 1.01–2.30, *p* = 0.044, Table 8) and after the onset of melasma (aOR = 1.69, 95% CI: 1.13–2.51, *p* = 0.010, Table 8). This confirms that kitchen exposure is a robust risk factor, independent of parity. In stark contrast, the significant associations previously observed for several other factors were substantially attenuated and rendered non‐significant after this adjustment. This included education level (*p* = 0.104), change of residence (*p* = 0.097), marital status (*p* = 0.809), and disease duration (*p* = 0.237) (Table 8). Similarly, the protective effect of electronics use, both before (*p* = 0.242) and after onset (*p* = 0.287), was no longer statistically significant (Table 8). These findings strongly suggest that the influence of these sociodemographic and lifestyle factors on vascular severity is largely mediated or confounded by parity. For outdoor exposure, a non‐significant trend was observed after adjustment, where shorter after‐onset exposure (≤ 2 h/day) tended to be associated with a higher risk compared to longer exposure (> 2 h/day) (aOR = 1.94, 95% CI: 0.90–4.20, *p* = 0.091, Table 8), suggesting a potential protective effect of longer outdoor activity that warrants further investigation. After adjusting for parity, the MEQ score emerged as a significant independent risk factor for more severe telangiectasia (aOR = 1.07, 95% CI: 1.00–1.13, *p* = 0.039, Table 8). This indicates that for each one‐point increase in the MEQ score (trending toward morningness), the risk of being in a more severe vascular category increases by 7%. In contrast, the categorical MEQ chronotype (e.g., Evening or Intermediate type vs. Morning type) did not show a significant association after the adjustment (*p* > 0.05 for both, Table 8).

**TABLE 8:** Multivariate ordinal logistic regression analysis of lifestyle factors (continuous) associated with telangiectasia severity, adjusted for parity (number of deliveries).

Variable/Parameter (per hour/day increase)	*B* (estimate)	SE	Adjusted OR (aOR)	95% CI for aOR	*p*
Change of residence (no vs. yes)	0.669	0.404	1.95	0.89–4.31	0.097
Education level (below bachelor vs. ≥ bachelor)	0.706	0.434	2.03	0.87–4.74	0.104
Marital status (unmarried vs. married)	0.282	1.169	1.33	0.13–13.11	0.809
Disease duration (years)	0.033	0.028	1.03	0.98–1.09	0.237
Electronics use (before onset, h/day)	−0.069	0.059	0.93	0.83–1.05	0.242
Electronics use (after onset, h/day)	−0.068	0.064	0.93	0.82–1.06	0.287
Kitchen exposure (before onset, h/day)	0.421	0.209	1.52	1.01–2.30	0.044
Kitchen exposure (after onset, h/day)	0.522	0.203	1.69	1.13–2.51	0.010
Outdoor exposure (before onset) ≤ 2 h/day (vs. > 2 h/day)	0.556	0.383	1.74	0.82–3.69	0.146
Outdoor exposure (after onset) ≤ 2 h/day (vs. > 2 h/day)	0.664	0.393	1.94	0.90–4.20	0.091
MEQ	0.063	0.03	1.07	1.00–1.13	0.039
MEQ type: Evening type	−0.662	1.066	0.52	0.06–4.17	0.534
MEQ type: Intermediate type	−0.456	0.545	0.63	0.22–1.85	0.403

### 3.4 Risk Factor Analysis for mMASI Score

To identify the independent factors associated with melasma severity, a multiple linear regression model was constructed with the mMASI score as the dependent variable. The overall model was statistically significant (*F*(18,112) = 4.534, *p* < 0.001) and explained 32.9% of the variance in mMASI scores (Adjusted *R* ^2^ = 0.329). Collinearity diagnostics indicated that there was no significant multicollinearity among the independent variables (all VIF < 5).

The detailed results of the regression coefficients are presented in Table 9. After adjusting for all covariates, four variables emerged as significant independent predictors of the mMASI score. The Melanin Index (MI) was identified as the most potent positive predictor (*B* = 0.032, 95% CI: 0.019 to 0.046, *p* < 0.001, Table 9). Conversely, three factors were found to be significant negative predictors. A longer duration of electronics use before onset was associated with a lower mMASI score (*B* = −0.373, 95% CI: −0.684 to −0.061, *p* = 0.019, Table 9). Intriguingly, after controlling for MI and other factors, a larger erythema area ratio also predicted a lower mMASI score (*B* = −0.075, 95% CI: −0.140 to −0.009, *p* = 0.026, Table 9). Furthermore, the timing of onset was identified as a significant independent predictor. Compared to patients whose melasma developed postpartum, those with a prepregnancy onset had a significantly lower mMASI score on average by 1.209 points (*B* = −1.209, 95% CI: −2.381 to −0.037, *p* = 0.043, Table 9).

**TABLE 9:** Univariate and multivariate analyses of factors associated with mMASI score.

Variable and category	Univariate analysis (spearman correlation) spearman's *r*	Univariate *p*	*B* (95% CI)	Multivariate analysis (linear regression) *p*	VIF
*Demographic and personal history*					
Age (years)	0.313	**0.0002**	0.012 (−0.113, 0.137)	0.85	3.176
BMI (kg/m^2^)	0.312	**0.0002**	0.312 (−0.043, 0.667)	0.084	1.59
Education level (below bachelor vs. ≥ bachelor)^a^	−0.261	**0.0021**	−0.61 (−2.363, 1.144)	0.492	1.523
Marital status (unmarried vs. married)^a^	0.225	**0.0083**	−1.523 (−4.325, 1.278)	0.284	3.888
Family history (no vs. yes)^a^	−0.174	**0.0419**	−0.439 (−1.791, 0.913)	0.521	1.148
Occupation (indoor vs. outdoor)^a^	0.211	**0.0132**	1.589 (−0.654, 3.833)	0.163	1.365
History of pregnancy (no vs. yes)^a^	0.209	**0.0145**	2.661 (−0.408, 5.73)	0.089	4.907
Age of onset (years)	0.220	**0.0097**	0.144 (−0.014, 0.303)	0.074	4.08
Timing of onset (before pregnancy vs. during pregnancy vs. postpartum)^a^	0.178	**0.0374**	−1.209 (−2.381, −0.037)	**0.043**	3.82
*Daily lifestyle habits*					
Electronics use (h/day), before onset	−0.151	0.0791	−0.373 (−0.684, −0.061)	**0.019**	2.719
Electronics use (h/day), after onset	−0.175	**0.0409**	−0.019 (−0.346, 0.309)	0.911	2.794
Kitchen exposure (h/day), before onset	0.239	**0.0050**	−0.006 (−0.926, 0.913)	0.989	2.512
Kitchen exposure (h/day), after onset	0.288	**0.0006**	0.587 (−0.395, 1.57)	0.239	2.435
*Objective skin parameters*					
Vascular type (per grade increase)	0.285	**0.0007**	0.469 (−0.367, 1.304)	0.269	1.606
Erythema area ratio (%)	0.304	**0.0003**	−0.075 (−0.14, −0.009)	**0.026**	1.944
MI	0.431	**< 0.0001**	0.032 (0.019, 0.046)	**< 0.001**	1.385
EI	0.171	0.0504	0.009 (−0.006, 0.023)	0.229	1.756
MEQ score	0.142	0.0974	0.005 (−0.102, 0.112)	0.925	1.336

*Note:* The multivariate linear regression model was adjusted for all variables listed in the table. Significant *p*‐values (*p* < 0.05) are highlighted in bold.

^a^ For categorical variables, Spearman's *r* indicates the correlation with mMASI, while the multivariate analysis shows the effect of the specified category compared to its reference group.

Additionally, while not reaching the threshold for statistical significance, BMI (*p* = 0.084), history of pregnancy (*p* = 0.089), and age of onset (*p* = 0.074) showed a trend toward being positive predictors of a higher mMASI score (Table 9).

## 4 Discussion

Melasma is a common and distressing pigmentary disorder, characterized by a complex pathogenesis in which the vascular component is increasingly recognized as a crucial factor for both its development and recurrence. Clinically, this vascular involvement often presents as erythema and telangiectasia. Advanced imaging techniques have provided compelling evidence for this association. For instance, studies using dynamic optical coherence tomography (d‐OCT) have revealed that increased vessel dilation and density are significant features of melasma lesions [6]. It is hypothesized that these vascular abnormalities contribute to the pathology by sustaining a chronic inflammatory and oxidative stress microenvironment, which in turn may disrupt the epidermal‐dermal junction and exacerbate hyperpigmentation [6].

Furthermore, quantitative analyses using photoacoustic microscopy (PAM) have demonstrated that the degree of vasodilation is closely related to the melasma subtype, highlighting the potential of objective vascular metrics to improve the precision of pre‐ and post‐treatment assessments [7]. These findings collectively establish vascular proliferation and dilation as a core component of melasma's pathophysiology. In recognition of this, the Chinese Expert Consensus on Melasma (2021) has already proposed a classification system that distinguishes between a pure melanized type (M‐type) and a mixed type with a prominent vascular component (M + V type) [8].

However, despite this growing acknowledgment of the vascular role, a detailed investigation into the correlation between the specific morphological patterns of telangiectasia and the severity of melasma remains scarce; moreover, a comprehensive analysis of the risk factors contributing to these distinct vascular patterns has not yet been reported. It is precisely this knowledge gap that our present study was designed to address.

This study provides a detailed analysis of the relationship between dermoscopically‐defined vascular patterns, skin pathophysiology, and a wide array of potential risk factors in melasma. Our findings not only confirm the crucial role of vascularity but also uncover complex interrelationships and novel associations that challenge conventional perspectives.

Our primary finding is that the severity of telangiectasia, classified by dermoscopy, is strongly and positively correlated with objective measures of erythema (Erythema Area Ratio, EI) and clinical severity (mMASI). This aligns with previous histopathological studies identifying increased vascularity as a hallmark of melasma lesions and supports the notion that vascular proliferation is intrinsically linked to the overall severity of the disease [9]. However, a nuanced picture emerged from our regression analyses. While univariate analysis identified mMASI and erythema parameters as risk factors for more severe vascular types, MI, surprisingly, was not. This suggests that while pigmentation and vascularity often coexist, the severity of pigmentation alone may not be the primary driver for the progression of vascular patterns.

One of the most intriguing findings of our study is the paradoxical relationship between vascular severity, skin barrier function, and skin structure. We observed that Trans‐Epidermal Water Loss (TEWL), a gold‐standard indicator of barrier impairment, decreased as vascular patterns became more severe, with the Reticular Type showing the lowest values. Concurrently, a significant increase in skin thickness was observed in the more severe types. This inverse relationship—where increased thickness is accompanied by decreased TEWL—raises a critical question: does this thickening represent a functional repair or a pathological alteration?

The relationship between skin thickness and TEWL is highly context‐dependent and varies across different dermatological conditions. In certain pathological states, skin thickening is paradoxically associated with a significant increase in TEWL, indicating a compromised barrier. A prime example is plaque psoriasis, where active lesions are markedly thickened but exhibit significantly higher TEWL values than healthy skin, showing a positive correlation between thickness and TEWL. This is attributed to proliferative but structurally defective keratinization and inflammation [10]. In contrast, under noninflammatory conditions of skin thickening, the correlation is typically negative. For instance, in a callus formed by chronic friction or pressure, the epidermal thickening is characterized by compact orthokeratosis. Here, fully keratinized cells and relatively intact intercellular lipid structures form a more robust physical barrier. Consequently, the TEWL value is usually lower than that of the surrounding normal skin, demonstrating enhanced barrier function and a negative correlation between thickness and TEWL.

This distinction between physiological and pathological thickening is crucial for interpreting our findings. We hypothesize that the situation in advanced melasma is distinct from both scenarios. We posit that the chronic inflammation and sustained vascular activity in severe melasma may trigger a pathological process of dermal remodeling and epidermal thickening. This thickened, perhaps fibrotic, tissue could then physically impede water loss, creating a facade of an improved barrier. Therefore, the observed “improvement” in TEWL in more severe types is likely a structural artifact rather than an indication of a healthier, functionally repaired barrier. This perspective challenges the traditional view that barrier dysfunction is a unidirectional cause of vascular dilation. Instead, our data suggest a more dynamic, bidirectional relationship where severe vascular changes in later stages may, in turn, induce a nonfunctional, structural remodeling of the skin. This would mean that the lower TEWL in advanced melasma does not reflect true barrier restoration but rather a less permeable, pathologically altered state.

Our investigation into demographic and lifestyle factors revealed several robust, independent risk factors for severe telangiectasia. After adjusting for confounders, older age, higher BMI, and prolonged kitchen/heat exposure consistently emerged as significant risks. The association with age is consistent with melasma being a manifestation of photoaging, where cumulative UV damage and “inflammaging” contribute to vascular changes [11]. Although the direct link between obesity and facial telangiectasia is not well established, we speculate this association may be mediated by systemic low‐grade inflammation or hormonal alterations, such as increased estrogen levels, which are known to be associated with higher BMI. The strong dose‐dependent risk of kitchen and heat exposure corroborates the role of thermal stimuli in exacerbating vasodilation.

Parity (number of deliveries) was identified as one of the most potent independent risk factors in our study, a finding that also highlighted its role as a major confounder in the interpretation of other variables. The significant risk associated with higher parity, complemented by the protective effect of nulliparity (no history of pregnancy), strongly implicates pregnancy‐related hormonal fluctuations in the pathogenesis of the vascular component of melasma. This aligns with the well‐documented effects on the pro‐angiogenic effects of estrogen [12]. Crucially, when we adjusted for parity, the significant associations previously observed for factors like education level, marital status, and change of residence were rendered non‐significant. This powerful finding suggests that many sociodemographic factors identified in univariate analysis are not independent risks, but rather surrogate markers for lifestyles and conditions associated with higher parity.

This confounding effect of parity was most evident in the case of electronics use. While univariate analysis suggested a surprising protective effect, this association was completely attenuated and lost its significance after adjusting for parity. Therefore, we hypothesize that longer electronics use is not biologically protective but it likely acts as a surrogate marker for a specific lifestyle or demographic profile (e.g., indoor occupation, younger age, and lower parity) which in turn is associated with less exposure to other established risk factors like heat and UV radiation.

A critical insight from our study pertains to the limitations of the mMASI score. In our multivariate model predicting mMASI, while the MI was, as expected, the strongest positive predictor, the erythema area ratio paradoxically emerged as a significant negative predictor. This finding likely represents a statistical suppression effect, suggesting that the mMASI scoring system is disproportionately weighted toward pigmentation intensity and concentration. We hypothesize that, for a given amount of melanin (MI), a more inflammatory phenotype (higher erythema) might correspond to a more diffuse, less concentrated pigmentation pattern. This diffuse pattern might be subjectively perceived by clinicians as less severe during an mMASI assessment compared to a phenotype with darker, more focal pigmentation on a less erythematous background. This observation underscores the potential limitations of relying solely on a single clinical score. It highlights the importance for clinicians to look beyond subjective scoring and integrate objective tools, such as dermoscopy and quantitative imaging, to distinctly evaluate the contributions of both the vascular and pigmentary components. Such a multifaceted assessment is vital for tailoring more precise and individualized treatment strategies for melasma.

The strengths of our study include the novel, detailed dermoscopic classification and the comprehensive analysis of a wide range of variables. However, we acknowledge several limitations. First, the cross‐sectional design inherently prevents the establishment of causal relationships; the associations we identified should be interpreted as correlations. Second, the assessment of some variables relied on patient recall, which may be subject to potential recall bias. Third, our exploratory approach involved a large number of univariate statistical comparisons, which increases the probability of Type I errors (false positives) due to multiple testing. We have sought to mitigate this by focusing our primary conclusions on the results of the multivariable regression models, and thus the univariate findings should be considered hypothesis‐generating. Fourth, although our sample size was substantial, it may still have had insufficient statistical power to detect weaker but potentially meaningful associations. Therefore, our findings should be validated in future large‐scale, multicenter, and longitudinal studies, which would also be crucial for elucidating the causal pathways underlying these associations.

In conclusion, our study establishes a refined dermoscopic framework for classifying the vascular component of melasma and identifies parity, higher BMI, and prolonged heat exposure as key independent risk factors for its severity. We also highlight the complex, often nonlinear relationships between lifestyle factors and the disease.

Critically, our findings provide critical insights into the interpretation of clinical scoring systems. We demonstrate that telangiectasia in melasma lesions is not merely present but is closely associated with fundamental skin remodeling, such as increased dermal thickness, likely signifying a state of persistent, low‐grade inflammation. This evidence suggests that the vascular component is a profoundly undervalued contributor to the overall clinical severity of melasma, especially when assessed by pigmentation‐centric scores like the mMASI. Therefore, we argue that the assessment of telangiectasia severity deserves greater attention in clinical practice. Moving forward, we hypothesize that therapeutic strategies aimed at reducing vasodilation and vascular proliferation may not only improve erythema but could also be a crucial prerequisite for facilitating the true restoration of a functional skin barrier.

Our findings pave the way for future research into the hormonal mechanisms driving vascular changes in melasma and advocate for a more integrated diagnostic and management approach that gives equal weight to both the vascular and pigmentary components of the disease.

## Author Contributions

Conceptualization: Hua Gu, Ying Tu, and Zhenghui Yang; methodology: Zhenghui Yang and Jing Wu; software: Zhenghui Yang; validation: Qiuyan Yao; formal analysis: Zhenghui Yang; investigation: Jing Wu, Zhenghui Yang, and Qiuyan Yao; resources: Hua Gu and Ying Tu; data curation: Zhenghui Yang and Jing Wu; writing – original draft preparation: Zhenghui Yang and Jing Wu; writing – review and editing: Hua Gu and Ying Tu; visualization: Zhenghui Yang; supervision: Hua Gu and Ying Tu; project administration: Jing Wu; funding acquisition: Hua Gu.

## Funding

This research was supported by Yunnan Revitalization Talent Support Program (Ying Tu), Yunnan Province Clinical Center for Skin Immune Diseases (2024YNLCYXZX0043), 535 Talent Project of First Affiliated Hospital of Kunming Medical University (2024535Q11).

## Ethics Statement

The study was conducted in accordance with the Declaration of Helsinki. The study protocol was approved by the Ethics Committee of the First Affiliated Hospital of Kunming Medical University. All participants provided written informed consent to participate in this study.

## Consent

The authors have nothing to report.

## Conflicts of Interest

The authors declare no conflicts of interest.

## Data Availability

The datasets used or analyzed during this study are available from the corresponding author on reasonable request.

## Endnotes

^1^
The ∆TIVI% was calculated as the difference between post‐ and pre‐stimulation TIVI values, divided by the pre‐stimulation TIVI value.
